# Supplementary material for: Development of a computer-aided design software for the quantitative evaluation of aesthetic damage
Source: PLoS One. 2019 Dec 18;14(12):e0226322. doi: 10.1371/journal.pone.0226322 (PMC6919621; doi:10.1371/journal.pone.0226322)
Supplement: S1 Code site — 1. (ZIP) [file pone.0226322.s001.zip › [EESC_jr][Projeto]Relatorio_07_21/reports/templates_zip/gentelella-master/vendors/Flot/examples/series-pie/index.html]

Flot Examples: Pie Charts


## Pie Charts

Default Options
Without Legend
Label Formatter
Label Radius
Label Styles #1
Label Styles #2
Hidden Labels
Combined Slice
Rectangular Pie
Tilted Pie
Donut Hole
Interactivity

### Source Code

  

## Pie Options

- **option:** *default value* - Description of option
- **show:** *false* - Enable the plugin and draw as a pie.
- **radius:** *'auto'* - Sets the radius of the pie. If value is between 0 and 1 (inclusive) then it will use that as a percentage of the available space (size of the container), otherwise it will use the value as a direct pixel length. If set to 'auto', it will be set to 1 if the legend is enabled and 3/4 if not.
- **innerRadius:** *0* - Sets the radius of the donut hole. If value is between 0 and 1 (inclusive) then it will use that as a percentage of the radius, otherwise it will use the value as a direct pixel length.
- **startAngle:** *3/2* - Factor of PI used for the starting angle (in radians) It can range between 0 and 2 (where 0 and 2 have the same result).
- **tilt:** *1* - Percentage of tilt ranging from 0 and 1, where 1 has no change (fully vertical) and 0 is completely flat (fully horizontal -- in which case nothing actually gets drawn).
- **shadow:**
  - **top:** *5* - Vertical distance in pixel of the tilted pie shadow.
  - **left:** *15* - Horizontal distance in pixel of the tilted pie shadow.
  - **alpha:** *0.02* - Alpha value of the tilted pie shadow.
  - **offset:**
    - **top:** *0* - Pixel distance to move the pie up and down (relative to the center).
    - **left:** *'auto'* - Pixel distance to move the pie left and right (relative to the center).
    - **stroke:**
      - **color:** *'#FFF'* - Color of the border of each slice. Hexadecimal color definitions are prefered (other formats may or may not work).
      - **width:** *1* - Pixel width of the border of each slice.
      - **label:**
        - **show:** *'auto'* - Enable/Disable the labels. This can be set to true, false, or 'auto'. When set to 'auto', it will be set to false if the legend is enabled and true if not.
        - **radius:** *1* - Sets the radius at which to place the labels. If value is between 0 and 1 (inclusive) then it will use that as a percentage of the available space (size of the container), otherwise it will use the value as a direct pixel length.
        - **threshold:** *0* - Hides the labels of any pie slice that is smaller than the specified percentage (ranging from 0 to 1) i.e. a value of '0.03' will hide all slices 3% or less of the total.
        - **formatter:** *[function]* - This function specifies how the positioned labels should be formatted, and is applied after the legend's labelFormatter function. The labels can also still be styled using the class "pieLabel" (i.e. ".pieLabel" or "#graph1 .pieLabel").
        - **radius:** *1* - Sets the radius at which to place the labels. If value is between 0 and 1 (inclusive) then it will use that as a percentage of the available space (size of the container), otherwise it will use the value as a direct pixel length.
        - **background:**
          - **color:** *null* - Backgound color of the positioned labels. If null, the plugin will automatically use the color of the slice.
          - **opacity:** *0* - Opacity of the background for the positioned labels. Acceptable values range from 0 to 1, where 0 is completely transparent and 1 is completely opaque.
        - **combine:**
          - **threshold:** *0* - Combines all slices that are smaller than the specified percentage (ranging from 0 to 1) i.e. a value of '0.03' will combine all slices 3% or less into one slice).
          - **color:** *null* - Backgound color of the positioned labels. If null, the plugin will automatically use the color of the first slice to be combined.
          - **label:** *'Other'* - Label text for the combined slice.
          - **highlight:**
            - **opacity:** *0.5* - Opacity of the highlight overlay on top of the current pie slice. Currently this just uses a white overlay, but support for changing the color of the overlay will also be added at a later date.

## Changes/Features

- *v1.0 - November 20th, 2009 - Brian Medendorp*
- The pie plug-in is now part of the Flot repository! This should make it a lot easier to deal with.
- Added a new option (innerRadius) to add a "donut hole" to the center of the pie, based on comtributions from Anthony Aragues. I was a little reluctant to add this feature because it doesn't work very well with the shadow created for the tilted pie, but figured it was worthwhile for non-tilted pies. Also, excanvas apparently doesn't support compositing, so it will fall back to using the stroke color to fill in the center (but I recommend setting the stroke color to the background color anyway).
- Changed the lineJoin for the border of the pie slices to use the 'round' option. This should make the center of the pie look better, particularly when there are numerous thin slices.
- Included a bug fix submitted by btburnett3 to display a slightly smaller slice in the event that the slice is 100% and being rendered with Internet Explorer. I haven't experienced this bug myself, but it doesn't seem to hurt anything so I've included it.
- The tilt value is now used when calculating the maximum radius of the pie in relation to the height of the container. This should prevent the pie from being smaller than it needed to in some cases, as well as reducing the amount of extra white space generated above and below the pie.
- **Hover and Click functionality are now availabe!**
  - Thanks to btburnett3 for the original hover functionality and Anthony Aragues for the modification that makes it compatable with excanvas, this was a huge help!
  - Added a new option (highlight opacity) to modify the highlight created when mousing over a slice. Currently this just uses a white overlay, but an option to change the hightlight color will be added when the appropriate functionality becomes available.- I had a major setback that required me to practically rebuild the hover/click events from scratch one piece at a time (I discovered that it only worked with a single pie on a page at a time), but the end result ended up being virtually identical to the original, so I'm not quite sure what exactly made it work.
    - Warning: There are some minor issues with using this functionality in conjuction with some of the other more advanced features (tilt and donut). When using a donut hole, the inner portion still triggers the events even though that portion of the pie is no longer visible. When tilted, the interactive portions still use the original, untilted version of the pie when determining mouse position (this is because the isPointInPath function apparently doesn't work with transformations), however hover and click both work this way, so the appropriate slice is still highlighted when clicking, and it isn't as noticable of a problem.
- Included a bug fix submitted by Xavi Ivars to fix array issues when other javascript libraries are included in addition to jQuery
  
- *v0.4 - July 1st, 2009 - Brian Medendorp*
- Each series will now be shown in the legend, even if it's value is zero. The series will not get a positioned label because it will overlap with the other labels present and often makes them unreadable.
- Data can now be passed in using the standard Flot method using an array of datapoints, the pie plugin will simply use the first y-value that it finds for each series in this case. The plugin uses this datastructure internally, but you can still use the old method of passing in a single numerical value for each series (the plugin will convert it as necessary). This should make it easier to transition from other types of graphs (such as a stacked bar graph) to a pie.
- The pie can now be tilted at an angle with a new "tilt" option. Acceptable values range from 0-1, where 1 has no change (fully vertical) and 0 is completely flat (fully horizontal -- in which case nothing actually gets drawn). If the plugin determines that it will fit within the canvas, a drop shadow will be drawn under the tilted pie (this also requires a tilt value of 0.8 or less).
  
- *v0.3.2 - June 25th, 2009 - Brian Medendorp*
- Fixed a bug that was causing the pie to be shifted too far left or right when the legend is showing in some cases.
  
- *v0.3.1 - June 24th, 2009 - Brian Medendorp*
- Fixed a bug that was causing nothing to be drawn and generating a javascript error if any of the data values were set to zero.
  
- *v0.3 - June 23rd, 2009 - Brian Medendorp*
- The legend now works without any modifications! Because of changes made to flot and the plugin system (thanks Ole Laursen!) I was able to simplify a number of things and am now able to use the legend without the direct access hack that was required in the previous version.
  
- *v0.2 - June 22nd, 2009 - Brian Medendorp*
- The legend now works but only if you make the necessary changes to jquery.flot.js. Because of this, I changed the default values for pie.radius and pie.label.show to new 'auto' settings that change the default behavior of the size and labels depending on whether the legend functionality is available or not.
  
- *v0.1 - June 18th, 2009 - Brian Medendorp*
- Rewrote the entire pie code into a flot plugin (since that is now an option), so it should be much easier to use and the code is cleaned up a bit. However, the (standard flot) legend is no longer available because the only way to prevent the grid lines from being displayed also prevents the legend from being displayed. Hopefully this can be fixed at a later date.
- Restructured and combined some of the options. It should be much easier to deal with now.
- Added the ability to change the starting point of the pie (still defaults to the top).
- Modified the default options to show the labels to compensate for the lack of a legend.
- Modified this page to use a random dataset. Note: you may need to refresh the page to see the effects of some of the examples.
  
- *May 21st, 2009 - Brian Medendorp*
- Merged original pie modifications by Sergey Nosenko into the latest SVN version *(as of May 15th, 2009)* so that it will work with ie8.
- Pie graph will now be centered in the canvas unless moved because of the legend or manually via the options. Additionally it prevents the pie from being moved beyond the edge of the canvas.
- Modified the code related to the labelFormatter option to apply flot's legend labelFormatter first. This is so that the labels will be consistent, but still provide extra formatting for the positioned labels (such as adding the percentage value).
- Positioned labels now have their backgrounds applied as a seperate element (much like the legend background) so that the opacity value can be set independently from the label itself (foreground). Additionally, the background color defaults to that of the matching slice.
- As long as the labelOffset and radiusLimit are not set to hard values, the pie will be shrunk if the labels will extend outside the edge of the canvas
- Added new options "radiusLimitFactor" and "radiusLimit" which limits how large the (visual) radius of the pie is in relation to the full radius (as calculated from the canvas dimensions) or a hard-pixel value (respectively). This allows for pushing the labels "outside" the pie.
- Added a new option "labelHidePercent" that does not show the positioned labels of slices smaller than the specified percentage. This is to help prevent a bunch of overlapping labels from small slices.
- Added a new option "sliceCombinePercent" that combines all slices smaller than the specified percentage into one larger slice. This is to help make the pie more attractive when there are a number of tiny slices. The options "sliceCombineColor" and "sliceCombineLabel" have also been added to change the color and name of the new slice if desired.
- Tested in Firefox (3.0.10, 3.5b4), Internet Explorer (6.0.2900, 7.0.5730, 8.0.6001), Chrome (1.0.154), Opera (9.64), and Safari (3.1.1, 4 beta 5528.16).

Copyright © 2007 - 2014 IOLA and Ole Laursen
